# A Case of Cancer of the Liver

**Published:** 1908-04-25

**Authors:** Wm. Hale White

**Affiliations:** Senior Physician and Lecturer on Medicine, Guy's Hospital.


### Hospital Clinics.

### A CASE OF CANCER OF THE LIVER.

By WM. HALE WHITE, M.D. Lond., F.R.C.P., Senior Physician and Lecturer on Medicine, Guy's Hospital.

Notes from a Clinical Lecture.

Gentlemen,—The case I am going to bring to your notice to-day is that of a woman lying in Miriam Ward. She is fifty-one years old. She came in on the 11th of this month for abdominal pain, sickness, and a tumour in the abdomen. She told us that her mother died of cancer, and that the present trouble has been of six weeks' duration. She says that it came on gradually with pain in the abdomen and back, and that she had had sickness daily since the commencement of the illness. She also says that she has lately lost weight, and her friends have noticed that she has been getting thinner for some time. When she came in, we observed that she looked older than her years, that she appeared wasted, that her skin was dry, inelastic, and faintly yellow. When we looked at the abdomen we saw an extraordinary state of things, for on the right side there was a large intra-abdominal lump that made the abdominal wall stand out. This lump was found to be hard; it filled the right loin, and it came upwards from there to the ribs, and extended downwards to some two inches below the umbilicus. Well, to make a long story short, in tracing it upwards we could not define it from the liver, and it moved up and down with respiration so that from its position and movement we had no doubt that it was an enlarged liver. It was found by percussion that the liver was enlarged upwards, but, as I have often pointed out to you, when this organ enlarges it does not often enlarge upwards, unless there is some local disease of it, which is likely to destroy the diaphragm. When there is a general enlargement of the viscus such as we see in a lardaceous or nutmeg liver, the enlargement is downwards. This patient's jaundice has now become quite definite; she has been frequently sick, bringing up large quantities of material which contains hydrochloric acid but no blood. She has very considerable leucocytosis, her temperature has remained normal, and that is really all I need tell you about her.

### The Causes of Irregular Enlargement of the Liver.

So you see the problem we have to decide about is, What possibly can be the cause of this great tumour? I have already pointed out that, from its position and from the fact that it moves up and down with respiration, and goes up under the ribs, it can hardly be anything else but an enlarged liver; but it is so gigantic, especially in the lower part, that at first sight it is quite pardonable to wonder whether it is so at all. The next thing to observe about it is that it is very irregular; there is a particularly large lump on the right lobe, and there are lumps along the lower border. The problem therefore is, What can possibly be the cause of a very large irregular liver?

Well now, the diseases that cause a very large irregular liver are five in number. First of all the patient may have carcinoma; secondly, cirrhosis; thirdly, abscess; fourthly, hydatid disease; or lastly, syphilitic disease. Any one of those five might be the cause of a large irregular liver. I think we had better take them in the reverse order, and first of all discuss whether she could by any possibility have syphilitic disease of the liver. In the first place it is uncommon. I have here the figures for the hospital showing the proportion of people who suffer from it. In twenty years we found syphilitic disease of the liver in the post-mortem room ninety-five times, a proportion of about 1 per cent., so you see it is uncommon. And then in more than half the people in whom, after death, the liver is found to be affected by syphilis the condition is not detected during life. Therefore syphilis of the liver is clinically far from common, and, further, is much less common in a woman than in a man. Its very rarity should make us think twice before diagnosing that condition. A liver affected with syphilis has in it gummata and large fibrous bands. These bands are often as wide as your finger, they cut the liver up into great areas, and they contract and make it irregular. Further, this irregularity is exaggerated by the deposition of gummata. This change rarely makes the liver as big as in our patient, but occasionally the syphilis produces also lardaceous disease, and then the organ may become very large and irregular. But I have already shown you that cases of syphilis of the liver are very few, so that a combination of lardaceous disease with fibrous bands and gummata would be very exceptional, and it is only by such a combination that a syphilitic liver could be as large as is that of our patient. Further, if she had lardaceous disease we should expect to find lardaceous disease of the spleen and the kidneys, but there is no evidence of either, so that for all these reasons we may conclude that our patient has not syphilitic disease of her liver. Then there is another point; people with hepatic syphilis are hardly ever jaundiced, but she is. We shall mention in a few minutes some points for supposing she has malignant disease, and they also tell against her having syphilitic disease.

Hydatid disease will cause an irregular surface, but the irregularities would be round and smooth. The irregularities on this are neither round nor smooth, so that it cannot be affected with hydatids, and so we are quickly able to exclude the possibility of hydatid disease.

Then again, abscess. It is quite true the somewhat high leucocytosis might make you expect pus; but she has no rise of temperature and no pain. A huge abscess would very likely make the skin tender and red, but then an abscess of the liver in anyone who has not been out of this country is quite exceptional. So we can put that out of our list.

And then the next question, you will remember, was: Has she got cirrhosis? Sir William Jenner used to say that nobody ever had cirrhosis as a cause of hepatic enlargement if the tangible lumps were bigger than a cherry. So that from the mere size of the lumps on her liver she can hardly be suffering from cirrhosis. Then again she has no ascites. So you see by a process of exclusion we are able to come to the opinion that this woman has malignant disease of the liver.

Now, malignant disease causes the greatest enlargement of the liver of any disease known. I had a patient in John Ward some years ago whose liver weighed 19 lbs., and I found one recorded by Dr. Christian in whom it weighed 33 lbs. No other disease causes the liver to be nearly so heavy. Malignant disease in this organ is very common. It is constantly turning up in the post-morten room, and a very considerable number of people who die, die from its effects. It is either primary or secondary, and the proportion of primary to secondary malignant disease is about 1 to 21.

### Characters of Cancer of the Liver.

There are a few points concerning malignant disease of the liver which I will tell you about, as they will be of use to you in helping to diagnose it. In the first place, it usually causes a large, hard, irregular lumpy feeling. Sometimes the growths have lasted long enough for some atrophy to take place in the centre, and consequently they become umbilicated like the umbilication of a cancerous breast. If you make a post-mortem on a long-standing case you will often notice that a great deal of the liver is destroyed. Sometimes there is a mere shell of hepatic substance with pounds of malignant disease in it. The wonder is that the patient lives so long. Sir Samuel Wilks was constantly saying that in many diseases when we see the state of affairs revealed in the post-mortem room the wonder is not that the disease killed the patient, but that it did not kill the patient long before. Certainly that is so with advanced malignant disease of the liver, and if this woman has it, the wonder is that she has not been dead long ago considering the enormous masses she has. The greater part of her liver must be destroyed. There is nothing more striking in the post-mortem room than some of these specimens of malignant disease, because of the variable and beautiful colouring. In the first place, there is very often jaundice, and, as a result, the tissue becomes stained like that of the other organs of the body, so that parts of it are bright yellow. Then again some of the cells undergo fatty change, so that there are patches of pale yellow. Often you may see four colours—the original colour, the bright yellow of jaundice, the dark purple of hæmorrhage, and the pale yellow as the result of fatty change. Sometimes the irritation of the growth leads to an excess of fibrous tissue. Not infrequently after a time the centre of growth becomes soft and shaggy, and very often under the tap in the post-mortem room you can wash parts of it away. I mentioned a moment ago that there is often hæmorrhage into the growth. That is rather important, because if the liver increases in size in the course of a few hours this is a strong argument in favour of growth. The sudden increase means hæmorrhage, and hæmorrhage into the liver hardly ever occurs except as the result of growth. When there is jaundice it is because the glands in the transverse fissure are enlarged from the deposition of new growth, and by pressing on the common duct they give rise to jaundice. But sometimes the jaundice is due to the fact that the small bile ducts themselves are pressed upon by masses of hepatic growth. It is no uncommon thing to see the growth growing through the walls of the veins, and so it sets up thrombosis of them. I need hardly add that often a hepatic growth forms adhesions to the surrounding structures. Sometimes the tumour grows along the round ligament up to the umbilicus, and it helps us very much in diagnosis if the umbilicus is hardened by the deposition of growth in it.

Turn for a moment to the gall-bladder. If the common duct is obstructed by a gall-stone, at the post-mortem you usually find the gall-bladder shrunk, the reason being that the gall-stone when it was in the gall-bladder set up chronic inflammation, and chronic inflammation means shrinkage. On the other hand, if the bile is kept back by pressure of a malignant growth on the common duct the gall-bladder is often dilated, being distended by bile. Half the people who have malignant disease of the liver show no evidence of it during life. Nor is that surprising, because the primary growth may kill the patient before the hepatic growth produces any symptoms. It is also easy to remember that in half the people in whom, during life, malignant disease here is detected, you cannot find the seat of the primary disease. Malignant disease of the liver is commoner in women than in men, the proportion being four women to three men. You should never give a diagnosis when growth is suspected until you have made the patient take a deep inspiration and expiration, because by so doing some mass under the ribs may be brought within range of your fingers. You would hardly believe that malignant disease of the liver may actually become so huge that it makes the abdomen bulge, but such is undoubtedly the case in the woman before you.

About half the people who have this trouble feel pain. This woman complains of pain, and it is very difficult to dissociate the pain of the malignant disease from the primary disease. About half the people get jaundice before they die, and something under a half get ascites. Sometimes the peritoneum of the abdominal wall or some adjacent viscus is affected by hepatic tumour touching some part of it, so that you get a secondary nodule exactly opposite to one in the liver.

We talked about jaundice in the last lecture, so we need not go into that now. But there is no disease, or hardly any disease, which leads to such a long lasting jaundice as you find with malignant disease of the liver. A patient with such a lesion may live for a year, and when jaundice has lasted a long time he or she becomes yellowish green, and a dark yellow green colour of the skin is a characteristic of the jaundice lasting a long time. A patient with such jaundice can hardly have anything but malignant disease. Often some right-sided pleural effusion occurs, because the malignant disease extends to the right pleura. Many people do not pay sufficient attention to the fact that malignant disease of the liver is frequently accompanied by a rise in temperature. Then you must remember that malignant disease often gives leucocytosis, and again produces very considerable wasting. A few months ago I saw a man who was rather young and somewhat wasted; he had a large liver, and it was lumpy; he had considerable temperature and considerable leucocytosis. The lumps were so irregular and so hard and unlike an abscess that there could be little doubt but that the liver was enlarged from the presence of new growth, although the temperature was so high and the leucocytosis so considerable as to suggest pus. Naturally his friends got desperate, and although most of the doctors who saw the patient thought he had malignant disease, yet on account of the high temperature and the leucocytosis the abdomen was at last opened, and then it was found, as most of us thought, that the patient had malignant disease.

With regard to the prognosis. Most people with malignant disease of the liver are dead within a year of the original diagnosis. Do not, please, think that your diagnosis is necessarily wrong if, after you have seen the patient, he begins to improve a little. What frequently happens is that patients have had a hard time outside the hospital; they have been struggling on with their work in spite of bad health, and when we put them to bed they rest, and are able to take food, and so for a little while they gain weight. Then unless you have some experience, you are inclined to throw up the diagnosis of malignant disease; but it is a good rule whenever you diagnose malignant disease to stick to it—you are more often right than wrong if you do. In our patient the liver is so enormous that there can be no doubt but that it is enlarged; but you must not forget that it may hastily be thought to be enlarged when really it is displaced downwards by tight lacing or from some other cause. Twice this year I have come across patients who have been wasted from diabetes, and whose liver was displaced downwards; in each case enlargement was suspected, and the wasting was ascribed to malignant disease. You must also be careful to make out that the enlargement is real. For instance, in the case of a tumour in the stomach, the stomach being so close to the liver may give the impression that the latter is enlarged. Sometimes, too, a tumour of the kidneys has been taken for one of the liver. You remember the reverse mistake was made in one of the wards last summer.

It may be a difficult matter to tell whether a patient has cancer or cirrhosis, but we have already mentioned most of the points of distinction; if the nodules are larger than a cherry the disease is not cirrhosis. If the nodules are umbilicated then, of course, the case is one of cancer. If they rapidly increase in size, then again it must be cancer. If the jaundice is dark green, the patient must have cancer. If the stools are clay-coloured you know it is cancer; but if the spleen is large you know the case is probably one of cirrhosis. On the other hand, if the gall-bladder is enlarged the case is probably one of malignant disease, for there is no particular reason why the gall-bladder should be enlarged in cirrhosis. You also have the history. These points, I think, will usually guide you aright. Sometimes it is very difficult to tell whether a patient has carcinoma of the liver or an enlargement from the presence of a gall-stone in the common duct. If the liver is enlarged irregularly the case is one of cancer; if there are rigors it is probably one of gall-stones. Never omit to search the stools for gall-stones. Sometimes gall-stones cause so much inflammatory thickening around the gall-bladder that the resemblance to malignant disease of the gall-bladder is very close.

This brings me to the point of what can be the seat of the primary disease of the patient in the ward, for we have seen that primary malignant disease of the liver is very rare. She has a tumour which is in such a position that it may be a malignant mass in the gall-bladder, or malignant disease of the pyloris; I really cannot say which, but this large mass makes me believe that the primary growth is in one or the other. But, on the other hand, she has had no history of gall-stones. It may interest you to know that among seventy-six cases of secondary cancer of the liver, twenty-four were due to primary growth in the stomach, and five only to growth of the gall-bladder, so you see the cause is far more likely to be in the stomach than the gall-bladder.

Just to round off the subject I might tell you the few facts we know about primary carcinoma of the liver. The older writers thought the proportion of secondary to primary malignant disease was five to one, but that is because they did not recognise primary malignant disease of the gall-bladder. There seem to be four well-defined groups of primary malignant disease of the liver. In the largest group, by far the commonest—64 per cent.—the primary malignant disease consists of scattered nodules; then in the next variety there is one great mass of tumour, that is called the massive variety; then there is the variety in which the growth is diffusely scattered; the fourth is a well-defined but rare variety, in which people with cirrhosis also have primary carcinoma. It is interesting to notice that primary malignant disease is commoner in men than in women, just the reverse of secondary. The frequency of malignant disease of the female pelvic organs and breast accounts for this difference. As you might expect, primary carcinoma of the liver is chiefly seen in adults; but there is a rare form occurring in children. I think in the cases I have observed the rise of temperature is rather commoner in primary malignant disease than in secondary. The primary form is much more rapidly fatal than the secondary. Patients with the primary form rarely live more than three months, so they rarely get deep jaundice because they do not live long enough.

